# Contact-electro-catalysis for the degradation of organic pollutants using pristine dielectric powders

**DOI:** 10.1038/s41467-021-27789-1

**Published:** 2022-01-10

**Authors:** Ziming Wang, Andy Berbille, Yawei Feng, Site Li, Laipan Zhu, Wei Tang, Zhong Lin Wang

**Affiliations:** 1 CAS Center for Excellence in Nanoscience, Beijing Institute of Nanoenergy and Nanosystems, Chinese Academy of Sciences, Beijing, 100083 China; 2 School of Nanoscience and Technology, University of Chinese Academy of Sciences, Beijing, 100049 China; 3 CAS Center for Excellence in Nanoscience, National Center for Nanoscience and Technology (NCNST), Beijing, 100190 China; 4 Department of Chemistry, Carnegie Mellon University, Pittsburgh, PA 15213 USA; 5 School of Materials Science and Engineering, Georgia Institute of Technology, Atlanta, GA 30332-0245 USA

**Keywords:** Electrocatalysis, Heterogeneous catalysis, Electrocatalysis

## Abstract

Mechanochemistry has been studied for some time, but research on the reactivity of charges exchanged by contact-electrification (CE) during mechanical stimulation remains scarce. Here, we demonstrate that electrons transferred during the CE between pristine dielectric powders and water can be utilized to directly catalyze reactions without the use of conventional catalysts. Specifically, frequent CE at Fluorinated Ethylene Propylene (FEP) - water interface induces electron-exchanges, thus forming reactive oxygen species for the degradation of an aqueous methyl orange solution. Contact-electro-catalysis, by conjunction of CE, mechanochemistry and catalysis, has been proposed as a general mechanism, which has been demonstrated to be effective for various dielectric materials, such as Teflon, Nylon-6,6 and rubber. This original catalytic principle not only expands the range of catalytic materials, but also enables us to envisage catalytic processes through mechano-induced contact-electrification.

## Abstract (web summary)

Contact electro catalysis (CEC) was proposed as a novel catalytic principle that uses electron transfers during contact electrification to accelerate chemical reactions. Here the authors show even catalytically inert pristine polymers can catalyze the degradation of organic pollutant.

## Introduction

Mechanochemistry was regarded as one of the ten changing world technologies by IUPAC,^1^ which generally relies on the increase of defects,^2–4^ local extreme conditions,^5–7^ or force-induced effects^8–10^ under external mechanical agitations to facilitate reactions. However, little attention has been paid to the potential contribution made by the contact electrification effect between liquid and solid, despite the frequent contacts and separations occurring during mechanochemical processes. Previous researches have demonstrated that organic pollutants can be degraded by magnetic stirring with piezoelectric materials, which is referred to as tribocatalysis.^11–13^ However, the utilization of piezoelectric materials in these studies actually makes the interpretation of the underlying mechanism ambiguous because of the presence of both piezoelectric and triboelectric effects. For example, the degradation mechanism by the same method has also been described as hydromechanics-induced piezocatalysis (Supplementary Table 1).^14–16^ To unambiguously understand the mechanism for general materials, solid evidence regarding the contributions made specifically by contact electrification to the chemical reactions are required. Pristine polymers represent an ideal choice for this purpose. Unlike conventional catalysts, such as metals,^17,18^ zeolites,^19,20^ semiconductors,^21,22^ and piezoelectric materials,^23,24^ pristine polymers, due to their stable electronic structures, have rarely been considered as suitable materials for catalysis (Supplementary Table 2). Recently, it has been demonstrated that dielectric materials can withdraw electrons from DI water during the contact electrification process, and intensive efforts have been directed towards the mechanism and application of liquid-solid CE in the meantime.^25–28^ Electrons were proved to contribute and in a majority of cases dominate the charge transfer process during CE at the water-dielectric interface,^29^ which implies that promoting reaction rate by CE should be possible.

Here, we first demonstrated that electrons exchanged during CE at the interface of water and dielectric powder could be utilized in chemical reactions, through a process called contact-electro-catalysis (CEC). The mechanism of CEC proposes that frequent contact-separation cycles at the surface of dielectric powder are induced by the growth and collapse of cavitation bubbles during mechano-stimulation,^30^ and electrons exchanged during such CE process could be transferred to different substrates to form reactive oxygen species (ROS). These CEC-yielded ROS in an aqueous solution can then react with refractory organic compounds in advanced oxidation processes (AOPs). The present study mainly focused on the degradation of a 5-ppm aqueous methyl orange (MO) solution in presence of 20 mg of Fluorinated ethylene propylene (FEP) powder. Liquid-chromatography mass-spectroscopy (LC-MS) results revealed that MO was completely degraded after 180 min of ultrasonication (40 kHz, 120 W). Ex-situ morphological and spectroscopic characterizations confirmed that both the physical and chemical properties of FEP powder remained unchanged after degradation. Besides, the electron paramagnetic resonance (EPR) verified the evolution of hydroxyl and superoxide radicals. DFT simulations have also been conducted to evaluate the energy barrier of electron exchange between FEP and water/O_2_ in such conditions. Contact-electro-catalysis (CEC), the catalysis of chemical reactions by CE driven electron exchange, standing at the frontier of mechanochemistry, CE, and catalysis, represents an innovative strategy for yielding ROS and dealing with refractory organic pollutants. A novel wastewater treatment system has been proposed on the basis of CEC principle due to its merits of scalability and recyclability, and we expect more promising reactive systems involving radical species could be established in the future, opening a new field for catalysis.

## Results

### Investigations on the degradation of methyl orange

**Fig. 1:**
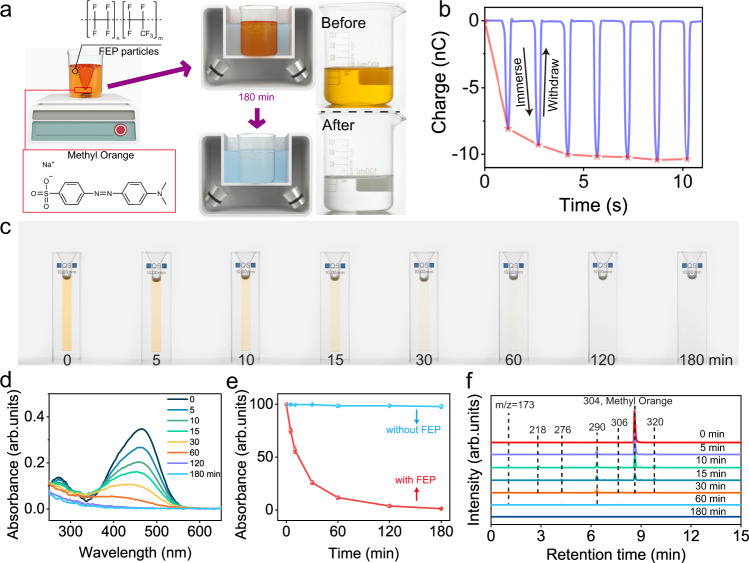
Degradation of methyl orange by contact-electro-catalysis. **a** 3D schematic of the experimental setup and protocol. **b** Measured electric output of a single electrode TENG that is repeatedly immersed in DI water. **c** Photographs of MO aqueous solution samples from 0 to 180 min. **d** UV-Vis spectra of a 50 mL aqueous methyl orange (MO) solution during ultrasonication in presence of FEP powder (20 mg) for 3 h. **e** Comparison of absorbance of MO solution between situations of with/without FEP powder. **f** Mass spectra of the MO solution after separation by liquid-chromatography. Error bars represent standard deviation based on three replicate data.

Figure 1 presents the systematic investigation conducted on the degradation of MO. Our experiment design is illustrated in Fig. 1a. 20 mg of FEP powder were added to a 50 mL aqueous solution of MO (5 ppm), and then stirred for 48 h to improve the contact between FEP and water. Thereafter, the as-prepared suspension was ultrasonicated at a frequency of 40 kHz and a power of 120 W. The original light-yellow solution becomes transparent after 3 h, as shown by inserted photos and Supplementary Movie 1. Ultrasonication is employed here for generating cavitation bubbles capable of inducing contact-separation cycles. The existence of contact electrification at the water-FEP interface is supported by the electrical output of a single electrode triboelectric nanogenerator (SE-TENG) that is repeatedly immersed in DI water (Fig. 1b). The magnitude of transferred charges for one single contact-separation cycle between water and FEP film increased from 8.05 nC to saturation at 10.47 nC, suggesting charges are induced and accumulated on the FEP surface during contact with DI water. The configuration of the entire setup is depicted in Supplementary Fig. 1. To investigate the discoloration process, aliquots (2 mL) were sampled at specific intervals and analyzed by UV-Vis spectroscopy. Corresponding optical photos and UV-Vis results are exhibited in Fig. 1c, d respectively. The characteristic absorbance peak of MO decreased as the ultrasonication time increased, and approached zero after 120 min. A control experiment has been conducted under the same condition except for the absence of FEP powder. However, no apparent diminution of absorbance was observed in this case, suggesting that the presence of FEP powder is a prerequisite to initiate the degradation process, see Fig. 1e. Afterward, liquid-chromatography mass-spectroscopy (LC-MS) was employed to identify the degradation products. Figure 1f depicts the chromatograms of a 5-ppm MO solution at different degradation times, and all peaks are labeled with corresponding mass-to-charge ratios *(m/z)*. The major peak at a retention time of 8.61 min, with an *m/z* of 304, corresponds to MO. Its intensity diminished along with the formation of other peaks, and all peaks disappeared after 180 min. Detailed analysis of the mass spectra (Supplementary Fig. 2) revealed that these peaks correspond to oxidative degradation products of MO, confirming the contribution from chemical reactions to the degradation. Although FEP is inert to a majority of chemicals, the surface charge density of FEP could reach around 50 μC/m^2^ after contact with water,^28^ and these charges are capable of contributing to chemical reactions.^31–33^ Therefore, various dielectric powders exhibiting different CE abilities, Polytetrafluoroethylene (PTFE), Polyvinylidene fluoride (PVDF), Nylon-66 (N6), and nitrile butadiene rubber (NBR) were employed here to investigate the relationship between degradation rate and CE properties (Supplementary Fig. 3). Differences in degradation rates are aligned with discrepancies in CE performances for particles that are negatively charged upon contacting water. FEP, exhibiting the highest surface charge density after contacting with water, is the best performer followed by PTFE and PVDF. It is noteworthy that positively charged particles, such as NBR and N6, show an overall lower rate of degradation than negative ones, and they all exhibit apparent coloration after degradation (Supplementary Fig. 4). This coloration is a consequence of adsorbing of MO at the surface, which can be ascribed to electrostatic attractions between the substrate and charged powder. For instance, MO, an anionic species, can be electrostatically adsorbed on the surface of these positively charged powders. This aggregation is unfavorable to degradation since the direct contact between water and these particles is hindered by accumulated MO, which is consistent with the divergence of degradation rate between positively and negatively charged powder. Degradation products of MO by these powders were also analyzed by LC-MS and available in Supplementary Fig. 5. The outperformance of FEP particles for the degradation of MO inspired us to further explore the essence of CEC.

### Characterization of the dielectric powders before/after the reaction

**Fig. 2:**
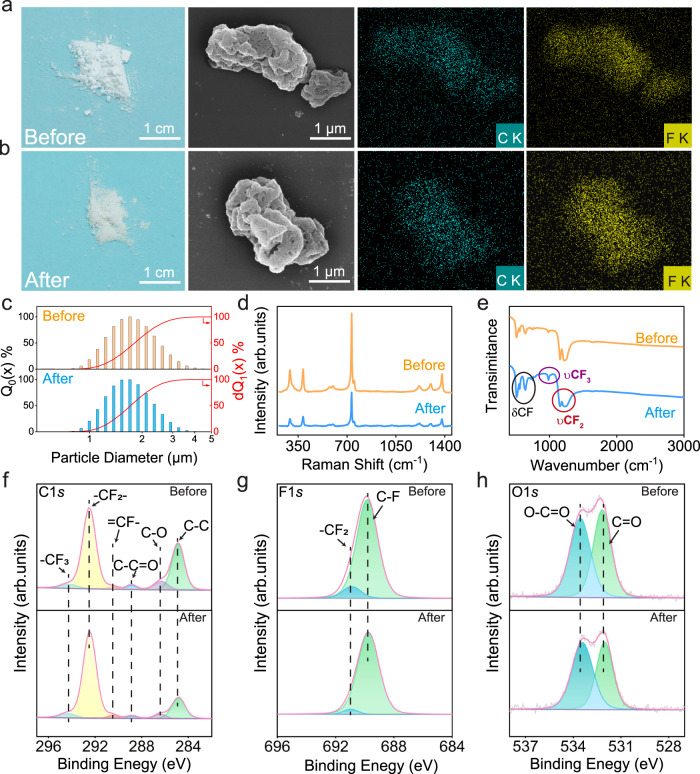
Characterization of FEP powder before and after contact-electro-catalysis. **a** Morphological characterization and energy dispersive X-ray (EDX) analysis of the FEP powder before reaction, as well as **b**, after the reaction. **c** Particle size distribution of FEP powder. **d** Raman spectra before (orange) and after (blue) the reaction. **e** Fourier Transform Infrared (FTIR) spectra before (orange) and after (blue) the reaction. **f** C1*s* **g** F1*s,* and **h** O1*s* XPS spectra of FEP powder before and after the reaction.

Morphological characterization and element mapping of FEP particles before and after degradation are reported in Fig. 2a, b. Neither obvious coloration nor modifications of the morphology was observed by the naked eye and scanning electron microscopy (SEM). Besides, the inserted mapping pictures indicate that the composition of FEP remained unchanged. A comparison of the particle size distributions before/after the experiment (Fig. 2c) proves that the FEP particles have neither aggregated nor decomposed during the experiment. Apart from morphological characterization, spectroscopic analysis techniques were also performed to deliver more in-depth information on the chemical properties of FEP powder. Figure 2d, e present Raman, and Fourier transform infrared (FTIR) spectroscopy results, respectively. In Raman spectroscopy, the skeleton vibration pattern of FEP before and after the experiment is identical. The fingerprint region in FTIR characterization, below 1500 cm^−1^, was also stable after the reaction. X-ray photoelectron spectroscopy (XPS) has been conducted to analyze the variation of the chemical state of FEP particles before/after the reaction. The C1*s*, F1*s*, and O1*s* spectra of the FEP powder are listed in Fig. 2f, h, respectively. Neither shift in binding energies of original peaks nor generation of new peaks was observed after degrading MO, which not only further confirms the chemical stability of FEP during CEC, but also excludes the possibility of physical adsorption of MO at FEP surface. These data indicate that the chemically inert FEP powder act as catalysts for the degradation of MO. In addition to the study of MO degradation, investigations on the degradation of Acid Orange-17 (AO-17) and Rhodamine B (RhB) by FEP particles were also proceeded. In the case of AO-17, an anionic dye, the observations were analogous to that of MO (Supplementary Fig. 6). However, the positive RhB ions were adsorbed on the surface of FEP after reaction (Supplementary Fig. 7), which is consistent with the hypothesis that physical adsorption is caused by electrostatic attraction between charged particles and substrates exhibiting reverse polarities, thus corroborating the hypothesis that contact electrification happens during the ultrasonication in presence of FEP.

### Generation of the reactive oxygen species (ROS)

**Fig. 3:**
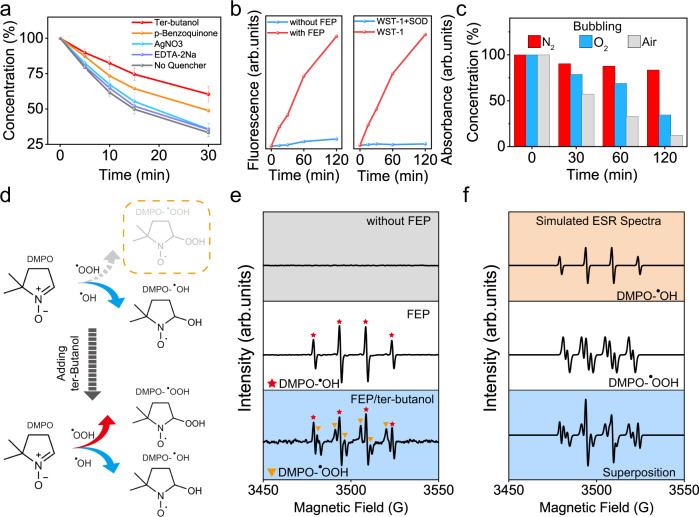
Investigation on the contribution of radicals to degradation of MO. **a** Evolution of MO concentration in conditions of various radical scavengers. Ter-butanol, p-benzoquinone, AgNO_3_, and EDTA-2Na, are regarded as superoxide radical, hydroxide radical, electron, and proton scavengers, respectively. **b** Evaluation of the production of OH and O_2_ radicals and their reactivities by the fluorescence intensity of THA-OH and the absorbance of formazan dye, respectively. **c** Investigation of the influence of dissolved gas in solution on the degradation of MO. **d** Schematic of the effect of introducing ter-butanol on the production of DMPO adducts. **e** Measured EPR spectra under various conditions, and every peak is labeled by corresponding patterns. **f** Simulated EPR spectra under the same condition by *EasySpin*. Error bars represent standard deviation based on three replicate data.

In order to further understand the underlying mechanism, a series of captures were added separately into the original solution, reaching a final concentration of 1 mM. The evolution of MO concentration in presence of these scavengers is displayed in Fig. 3a. The results indicated that two kinds of radicals, hydroxyl radicals (·OH) and superoxide radicals (·O_2_^−^), contribute to the degradation of MO. The hydroxyl radical appeared as the limiting factor as only 39.56% of MO was degraded after 30 min when it was quenched. The production of reactive radicals was estimated by terephthalic acid (THA) and a water-soluble tetrazolium salt (WST-1) experiment. As depicted in the left panel of Fig. 3b, the emission intensity of THA-OH adduct (425 nm) increased by 14-fold over reaction time in presence of FEP powder (Supplementary Fig. 8). Moreover, as depicted in the right panel, the peak of formazan dye (450 nm) does not appear during the WST test after introducing superoxide dismutase (SOD). (Supplementary Fig. 9). The relationship between the concentration of dissolved O_2_ and the degradation rate was further explored by constantly bubbling air, N_2_, or O_2_ (Fig. 3c). The fastest degradation was achieved while bubbling air (87.6%), and the lowest when bubbling N_2_ (16.5%). It is noteworthy that saturating the solution with O_2_ also hindered the degradation, with the removal of 65.5%. This is mainly attributed to the fact that oxidative atmospheres are detrimental to the contact electrification properties of materials, thus less electrons were induced and transferred during CEC.^34^ Electron paramagnetic resonance spectroscopy (EPR) was also carried out to confirm the production of ·O_2_^-^ and ·OH radicals. Two protocols were employed here: a 100 mM DMPO solution, as well as a solution containing both 100 mM DMPO and 1 mM ter-butanol. Ter-butanol was utilized to quench ·OH radicals, enhancing the opportunities for superoxide radicals to react with DMPO, as illustrated by Fig. 3d. No visible peak was measured during ultrasonication without FEP powder, as depicted by the top section of Fig. 3e. In contrast, the curve on the white background infers that quadruplet DMPO-·OH characteristic peaks were yielded when ultrasonication was applied in presence of FEP particles. (Supplementary Fig. 10) And sextuplet DMPO-·OOH peaks were not detected until introducing 1 mM ter-butanol (blue background). The final profile was a superposition of both quadruplet DMPO-·OH peaks and sextuplet DMPO-·OOH peaks, labeled by red stars and orange triangles respectively. The entire profile is dominated by a quadruplet DMPO-·OH peak, which can be ascribed to the fact that hydroxyl radicals are more prone to react with DMPO and that the hydroxyl adduct is more stable than that of superoxide radicals.^35^ Simulated ESR spectra of individual hydroxyl and superoxide radicals, as well as their superposition with respective weights of 70 and 30%, are displayed in Fig. 3f. Raw code for simulation is listed in Supplementary Note 1. The calculated EPR spectra are consistent with acquired data in Fig. 3e, and divergences between them are mainly due to the nonequilibrium status during the measurement. (Supplementary Fig. 11)

### Mechanism of contact-electro-catalysis

**Fig. 4:**
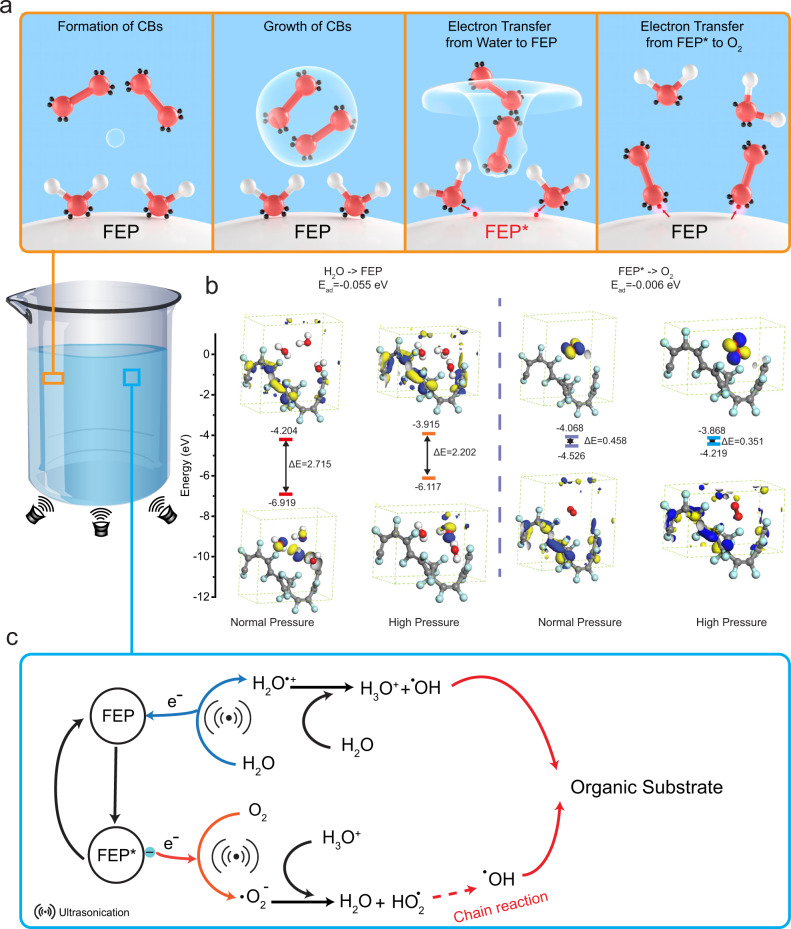
Mechanism of contact-electro-catalysis. **a** Schematic representing the contact-electro-catalysis phenomenon during ultrasonication. The red sphere represents the oxygen atom, while the white for a hydrogen atom, and black for electrons. **b** DFT calculations of the values of LUMO and HOMO levels for Water-FEP and O2-FEP in various conditions as indicated by the legend. **c** Proposed mechanism for the degradation of MO by contact-electro-catalysis generated radicals.

Contact-electro-catalysis (CEC) was proposed as the mechanism for degrading MO in presence of FEP particles. The propagation of ultrasonic waves in solution provokes the formation of cavitation bubbles (CB). The collapse of cavitation bubbles is assumed to induce frequent contact electrification at the FEP-water interface, from which arises electron exchanges. The step-by-step illustrations are exhibited in Fig. 4a. A nucleus of CB is firstly formed during ultrasonication. Thereafter, the CB, containing dissolved gas, grows from the nucleus until reaching a critical size. At this point, the collapse of the CB creates a high-pressure microjet that chases the previously adsorbed water molecules on the FEP surface. An electron is transferred from water to FEP upon contact, and the notation of FEP* is proposed to describe the charged state of FEP after separation from water. In the meantime, the enclosed O_2_ is released and grabs the electron from the charged surface of FEP* once they collide. FEP* retrieves its initial uncharged state after exchanging this electron to O_2_, and this cycle repeats itself as long as the emission of ultrasonic waves is sustained. The energy barriers for realizing these electron exchanges processes were assessed by Density Functional Theory (DFT). The specific calculation method is available in Supplementary Note 2. Considering CE is sensitive to external conditions, the high-pressure environment resulting from the collapse of the CB was also taken into consideration. In cases of water/FEP and FEP^*^/O_2_, calculations presented in Fig. 4b reveal the energy barriers for electron transferring in these two scenarios decreased by 18.8% and 23.3% respectively. Therefore, the formation and collapse of CBs could not only induce contact-separation cycles but also facilitate electron transfers during CE. Figure 4c wraps up and illustrates the potential mechanism for degrading organic pollutants by CEC. On the one hand, single electron transfer (SET) between water and FEP during CE results in the formation of water radical cations. Thus generated water radical cations undergo a rapid proton transfer from water, forming hydronium cations and hydroxyl radicals^36^. On the other hand, electrons accumulated at FEP^*^ surface are captured by O_2_, forming ·O_2_^−^ radicals. Then, ·O_2_^−^ are protonated into hydroperoxyl (HO_2_^·^),^37^ leading to the formation of hydroxyl radicals by a chain reaction. Hydroxyl radicals generated at the end of both steps then react with organic pollutants in an aqueous solution.

### Scalability and recyclability of the contact-electro-catalysts

**Fig. 5:**
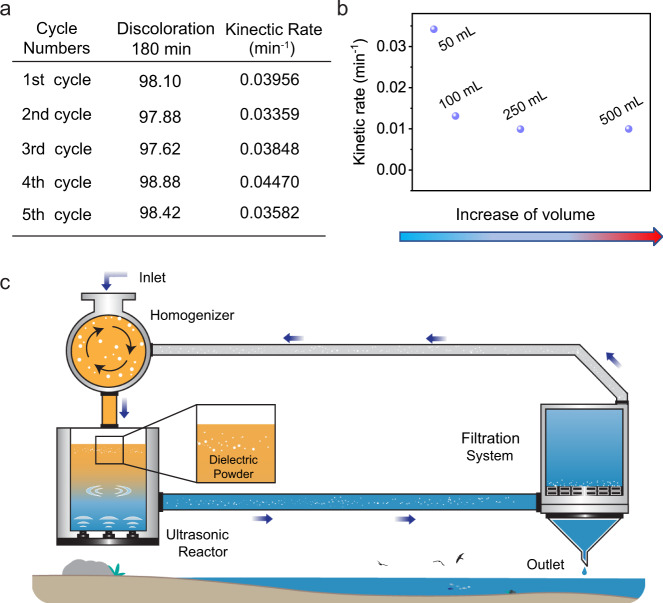
Recyclability of contact-electro-catalysts. **a** Evolution of degradation from one to five cycles of reaction. **b** Investigations on the degradation rate for different volume MO solutions. **c** Expected large application of contact-electro-catalysis for the treatment of organic wastewater.

No obvious diminution in the performances for the degradation of MO has been observed after recycling the FEP powder 5 times, as seen in Fig. 5a. Besides, this catalytic strategy can also be scaled up as exhibited in Fig. 5b. A slight decrease in kinetic constants for larger beakers is mainly due to the mismatch between ultrasonication power and the volume of solutions. Moreover, owing to the CE phenomenon’s ubiquitous existence, a broad range of materials could be utilized to degrade organic pollutants based on CEC, and these materials are generally commercially available and inexpensive polymers. A compact architecture was devised for the cost-effective treatment of organic wastewater on the basis of the contact-electro-catalysis principle, as demonstrated in Fig. 5c. And we expect this attractive strategy to be a promising candidate for applications in chemical engineering, biological research, and fields that are closely related to ROS.

## Discussion

A unique catalytic principle, contact-electro-catalysis (CEC), was first proposed and systematically investigated, which employs surface polarized electrons induced by contact electrification to accelerate chemical reactions. Ultrasonication-induced cavitation bubbles could not only generate the contact-separation cycles but also facilitate electron transfer by decreasing energy barriers for various active species generation. Our results indicated a 50 mL 5-ppm MO aqueous solution was completely degraded after 3 h of ultrasonication in presence of 20 mg of pristine FEP powder. And this catalytic efficiency could be further enhanced by introducing micro-nano structures on dielectric powder to increase the contact surface area or by chemical modifications as a mean to improve the surface charge density. As a ubiquitous phenomenon among various interfaces, contact electrification endows its derived contact-electro-catalysis with the power to greatly enrich the category of catalytic mechanisms and broaden the range of materials to be regarded as catalysts.

## Methods

### Chemical reagents

Methyl orange [C_14_H_14_N_3_NaO_3_S, Macklin, 98%], acid orange-17 [C_18_H_15_N_2_NaO_4_S, Macklin], rhodamine B [C_28_H_31_ClN_2_O_3_, Macklin, 99%], p-benzoquinone [C_6_H_4_O_2_, Macklin 99.5%], p-phthalic acid [C_8_H_6_O_4_, Macklin, 99%], sodium phosphate tribasic dodecahydrate [Na_3_PO_4_, Aladdin, 99.99%], ethylenediaminetetraacetic acid disodium salt dihydrate [C_10_H_14_N_2_Na_2_O_8_·2H_2_O, Aladdin, 99%], tert-butanol [C_4_H_10_O, Sinopharm Chemical Reagent Co., Ltd, 98.0%], silver nitrate [AgNO_3_, 99.8%, Sinopharm Chemical Reagent Co., Ltd], 5,5-dimethyl-1-pyrroline *N*-oxide [C_2_H_6_OS, Dojindo], WST assay Kit S311 [Dojindo], superoxide dismutase from bovine [S5395-30KU, Sigma-Aldrich], potassium bomide [KBr, 99.997%, Aladdin], fluorinated ethylene propylene (FEP) [Dupont], polytetrafluoroethylene (PTFE) [Dupont], polyvinylidene fluoride (PVDF) [(C_2_H_2_F_2_)n, SOLVAY], nylon-6,6 [(C_12_H_22_N_2_O_2_)n, Dupont], nitrile butadiene rubber (NBR) [Kumho], and AlN from Macklin.

### Sample preparation

A 5-ppm aqueous methyl orange solution was prepared by adding 5 mg of C_14_H_14_N_3_NaO_3_S in 1 L of ultrapure water, followed by magnetic stirring for 1 h.

About 20 mg of polymer powder were added into a beaker containing 50 mL of the as-prepared methyl orange solution, and then magnetically stirred at 1000 rpm for 48 h. The solution containing MO and the powder was ultrasonicated (40 kHz, 120 W) using an ultrasonic bath (Yumeng, YM020S). Aliquots were sampled at 0, 5, 10, 15, 30, 60, 120, 180 min. The temperature in the ultrasonic bath was regulated.

The solution of terephthalic acid was prepared by adding 332.4 mg of p-phthalic acid and 760 mg of sodium phosphate tribasic dodecahydrate.

The WST-1 solution was prepared by diluting 1 ml of WST-1 solution from a DOJINDO assay kit, into 19 mL of buffer solution and adding 30 mL of ultrapure water.

The powders after reactions were separated from the solution using a vacuum filtration system. The filtered powders were then dried in an oven at 40 degrees overnight before analysis.

FTIR samples were prepared by grinding 0.5 mg of polymer powders with 100 mg of KBr and then pressing them into a pellet.

Samples for EPR analysis were prepared by stirring 50 mL of ultrapure water and 20 mg FEP powders for 48 h at 1000 rpm. About 0.5 mL of DMPO was transferred to the solution and stirred for 5 min at 500 RPM prior to ultrasonication.

The MO solution for the scalability assessment was prepared as described above. The quantity of FEP powder was added proportionally to the volume of the solution treated. For instance, to treat a solution of 200 mL, 80 mg of FEP powder were added.

### Sample characterization

The UV-Vis absorbance of the aliquots was measured using a Shimadzu UV-3600 UV-Visible spectrometer on a range of 250–650 nm. The samples were placed into a Hellma Analytics QS High precision cell (Art. No. 104-10-40), with a light path of 10 mm.

The emission spectra of THA-OH were measured on an Edinburgh Instruments FLS 980, using λ_excitation_ = 225 nm and λ_emission_ = 425 nm.

The Scanning electron microscopy (SEM) images of the samples were obtained using an FEI Nova 450.

The Energy Dispersive X-Ray analysis (EDX) were conducted on FEI Nova 450 equipped with an AMETEK Octane Super appendix.

The X-ray photoelectron spectroscopy measurements have been conducted on a Thermo Fisher Scientific K-Alpha, in a vacuum of 1 × 10^−9^ mBar, using an Alka ray source (hv = 1486.6 eV), the working voltage is 15 kV and the filament current is 10 mA. The signal accumulation was performed for five to ten cycles. The pass energy is set at 30 eV.

The LC-MS analysis were conducted using a Thermo Scientific Q Exactive Orbitrap Quadrupole-Electrostatic Field Orbitrap High-Resolution Tandem Mass Spectrometer. The HESI ion source of the mass spectrometer was set at −3.0 kV, in positive ion mode. The mass spectrometry scanner was set on the full scan range of 100–1000 m/z. The resolution of the instrument is 70000 FMHM. The column used was a Hypersil Gold C18 (2.1 × 100 mm, 1.9 μm), the column temperature is set at 40 °C. The injection volume is 5 μL. Mobile phase A is composed of 0.1% formic acid aqueous solution, and mobile phase B is an acetonitrile solution.

The Raman spectroscopy analysis was conducted on a LabRam HR evolution (HORIBA, SAS France), using a range from 300 to 1400 cm^−1^.

FTIR analysis were conducted on a Bruker Vertex 80 v on a range from 400 to 3000 cm^−1^.

Electron paramagnetic resonance were recorded on a Bruker EMX plus-9.5/12/P/L. The measurements were conducted in X-Band (9.830243 GHz), with amplitude modulation of 1 G, microwave power of 2 mW, and an amplitude modulation frequency of 100 kHz and conversion time of 60 ms, and a time constant at 40.96 ms. The assignment of the components of the spectra was based on literature and simulation using *Easyspin*. The code used for the simulation of the spectra is presented in Supplementary Note 1.

## Supplementary information

Supplementary Information

Description of Additional Supplementary Files

Supplementary Movie 1

## Data Availability

The data supporting the findings of this study are reported in the main text or the Supplementary Information. Raw data can be obtained from the corresponding authors upon reasonable request.
