# Supplementary material for: Contact-electro-catalysis for the degradation of organic pollutants using pristine dielectric powders
Source: Nat Commun. 2022 Jan 10;13:130. doi: 10.1038/s41467-021-27789-1 (PMC8748705; doi:10.1038/s41467-021-27789-1)
Supplement: Supplementary file 1 — Supplementary Information [file 41467_2021_27789_MOESM1_ESM.pdf]

Supplementary Materials for

Contact-electro-catalysis for the Degradation of Organic Pollutants Using Pristine Dielectric Powders

Ziming Wang[1,2,†], Andy Berbille[1,2,3,†], Yawei Feng[1,2], Site Li[4], Laipan Zhu[1,2], Wei Tang[1,2,*], and Zhong Lin Wang[1,2,5,*]

[1] CAS Center for Excellence in Nanoscience, Beijing Institute of Nanoenergy and Nanosystems, Chinese Academy of Sciences, Beijing, 100083, China.

[2] School of Nanoscience and Technology, University of Chinese Academy of Sciences, Beijing, 100049, China.

[3] CAS Center for Excellence in Nanoscience, National Center for Nanoscience and Technology (NCNST), Beijing 100190, China

[4] Department of Chemistry, Carnegie Mellon University, Pittsburgh, Pennsylvania 15213, USA

[5] School of Materials Science and Engineering, Georgia Institute of Technology, Atlanta, GA 30332-0245, USA.

[†] These authors contributed equally to this work.

[*]Corresponding author. Email: tangwei@binn.cas.cn (W.T.); zhong.wang@mse.gatech.edu (Z.L.W.).

The PDF file includes:

Supplementary Note 1. Details of EPR simulations.

Supplementary Note 2. Details of DFT simulations.

Supplementary Fig. 3 | Evolution of UV-Vis absorbance of the MO solution during ultrasonication with PTFE, PVDF, Nylon-6,6 and NBR Rubber, respectively.

Supplementary Fig. 4 | Photographs of various dielectric powders before and after degradation of MO. (PTFE and PVDF are negatively charged powders, while Nylon-6,6 and NBR are positively)

Supplementary Fig. 5 | Mass spectra of a 5-ppm MO solution during degradation by various powders.

Supplementary Fig. 6 | Investigation on the discoloration of Acid Orange 17 (AO-17) by CEC using FEP powders.

Supplementary Fig. 7 | Investigation on the discoloration of Rhodamine B (RhB) by CEC using FEP powders.

Supplementary Fig. 8 | Investigations on the generation of reactive hydroxyl radicals.

Supplementary Fig. 9 | Investigations on the generation of reactive superoxide radicals.

Supplementary Fig. 10 | Evolution of EPR diagrams in absence/ presence of FEP powders under ultrasonication.

Supplementary Fig. 11 | Evolution of EPR diagrams after introducing 1 mM ter-butanol.

Supplementary Table 1. Comparisons between representative tribocatalysis literatures and this work

Supplementary Table 2. Main features of conventional catalysts for degrading pollutants under ultrasonication and their comparisons with this work.

Other Supplementary Materials for this manuscript include the following:

Supplementary Movie 1-Video demonstration of degrading MO solution in presence of pristine FEP powder, and its comparison to without FEP.

Supplementary Note 1:

EasySpin with version of 5.2.30 was employed to simulate the waveform of hydroxyl and superoxide radicals.[1] The raw code is listed as follows:

Easy spin spectra simulation:

```
clear, clc,
[x0, y0, pars] = eprload('test.dsc');
y01 = y0/(max(y0)-min(y0));
Exp.mwFreq = 9.830243;
Exp.Range = [min(x0) max(x0)]*0.1;

Sys1.g = 2.0056;
Sys1.Nucs = '1H, 14N';
Sys1.n = [1 1];
Sys1.A = [14.9 14.9]*2.8;
Sys1.lw = 0.14;
[x1,y1] = garlic(Sys1, Exp);
y10=y1/(max(y1)-min(y1));

Sys2.g = 2.0058;
Sys2.Nucs = '1H, 14N, 1H';
Sys2.n = [1 1 1];
Sys2.A = [10.5 13.5 2]*2.8;
Sys2.lw = 0.2;
[x2,y2] = garlic(Sys2, Exp);
y20=y2/(max(y2)-min(y2));
```

Supplementary Note 2:

In the present work, density functional theory (DFT) calculation was performed employing the Dmol[3] code.[2] Owing to the weak-coupling limit, i.e., a negligeable overlap of the molecular orbital, the difference between the LUMO of the acceptor and the HOMO of the donor is considered as equivalent to the charge transfer energy.[3] The exchange-correlation interaction was treated using the generalized gradient approximation (GGA) with PBE functional.[4] A double numerical quality basis set with d-type polarization function (DNP)[5] was utilized for all the geometric optimizations, total energy calculations. The core electrons were modeled by effective core pseudopotentials (ECP) by Dolg[6] and Bergner[7]. All the calculations were spin unrestricted. The conductor-like screening model (COSMO) with dielectric constant of 78.54 was used to simulate a solvent environment for the calculation. The effect of the Electric Double Layer has been neglected. The positions of all the atoms were fully relaxed until the following convergence criterion were met respectively: 0.002 Ha/Å for force, 10−5 Ha for total energy and 0.005 Å for displacement. The real space cutoff radius was 4.1 Å. The self-consistent field computations criterion was set at 10^{-6} Ha.

The pressure induced by cavitation bubbles is around 100 MPa[8], and the pressure required for compressing FEP to 5% extent is 15.2 MPa according to the datasheet provided by DuPont. The FEP micro-particles are regarded to undergo elastic compression in the calculation since micro-particles of polymers can behave elastically beyond the linear region. This assumption is also supported by several experimental observations, such as the study of the morphology (by SEM in Fig. 2a) and the evaluation particle size distribution (by particle size analyzer in Fig. 2b) of FEP particles before and after reaction. Therefore, the actual compressive ratio of FEP particles R_c under 100 MPa pressure can be expressed as:

$$R_c = \frac{100}{15.2} \times 5\% = 32.89\% \qquad (1)$$

For the simplicity of calculations, the compressive ratio of 30% was employed for DFT simulations. The compression is assumed to occur along with the folding direction of FEP molecular chains which is susceptible to external pressures. As a consequence, the compression of volume from 10 ×10×5 Å to 10×7×5 Å is regarded as the equivalent to the result of the high-pressure environment induced by the collapse of cavitation bubbles.

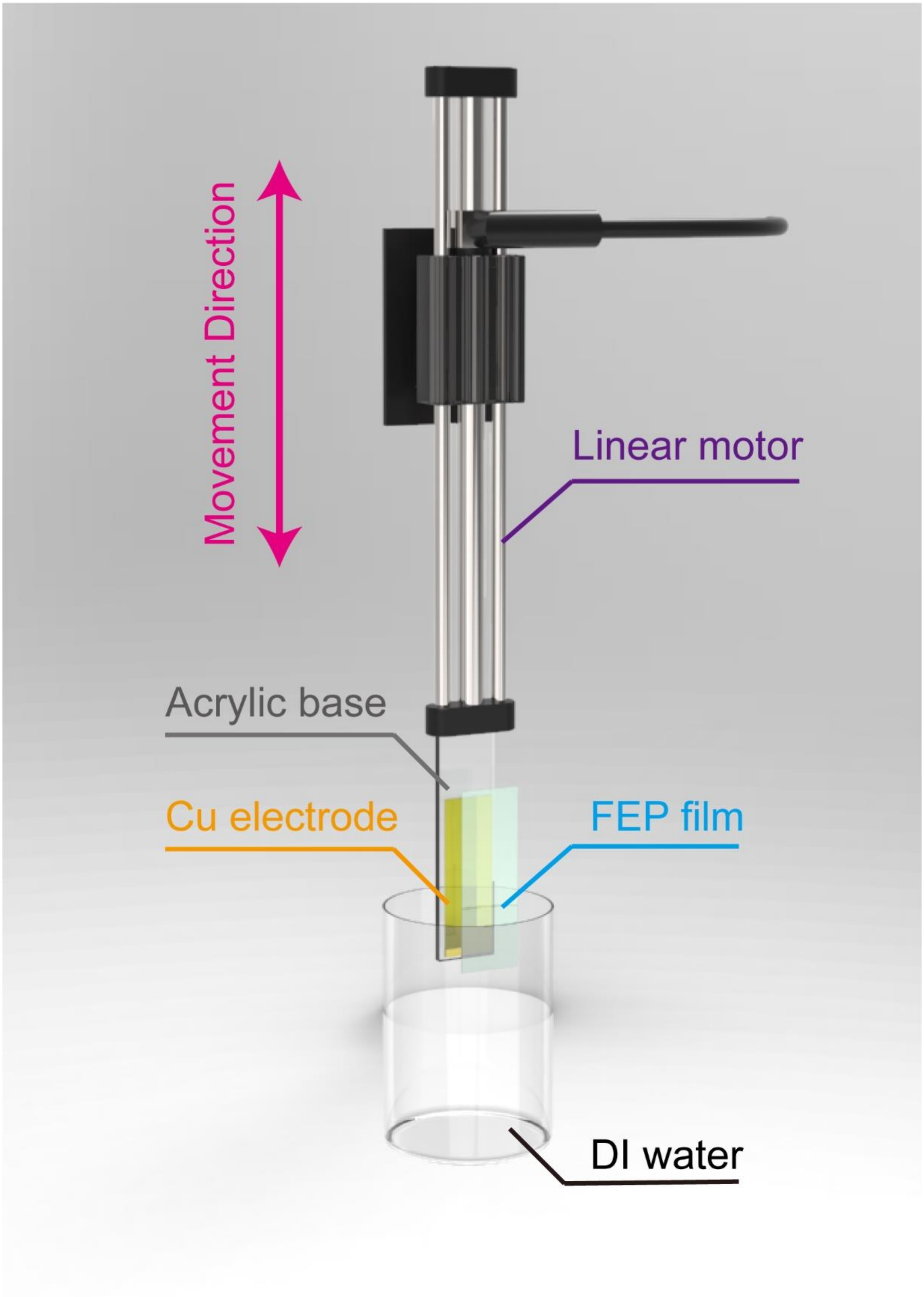

Supplementary Fig. 1 | Configuration of measuring the transferred charge of a SE-TENG that is repeatedly immersed in DI water.

m/z	173	218	276	290	306	320
**Degradation Products**	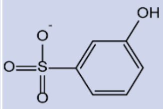	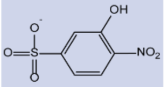	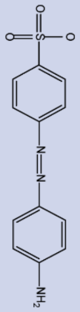	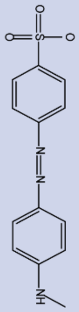	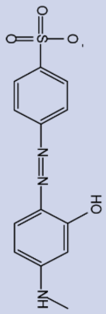	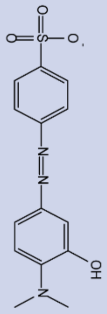

Supplementary Fig. 2 | The chemical structure of the product during the CEC degradation of methyl orange as identified by mass-spectroscopy.

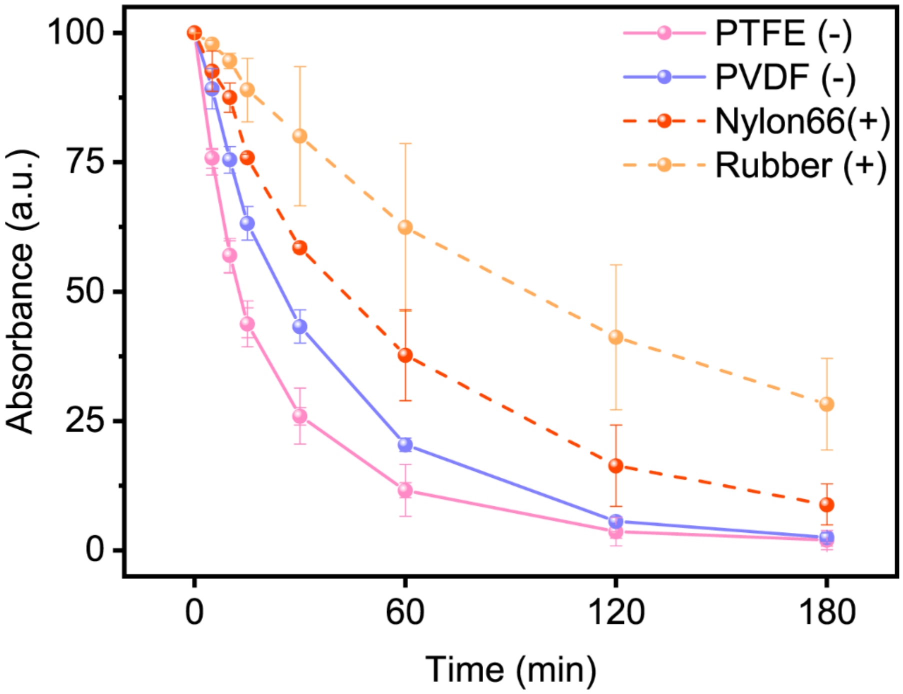

Supplementary Fig. 3 | Evolution of UV-Vis absorbance of the MO solution during ultrasonication with PTFE, PVDF, Nylon-6,6 and NBR Rubber, respectively. Error bars represent standard deviation based on three replicate data.

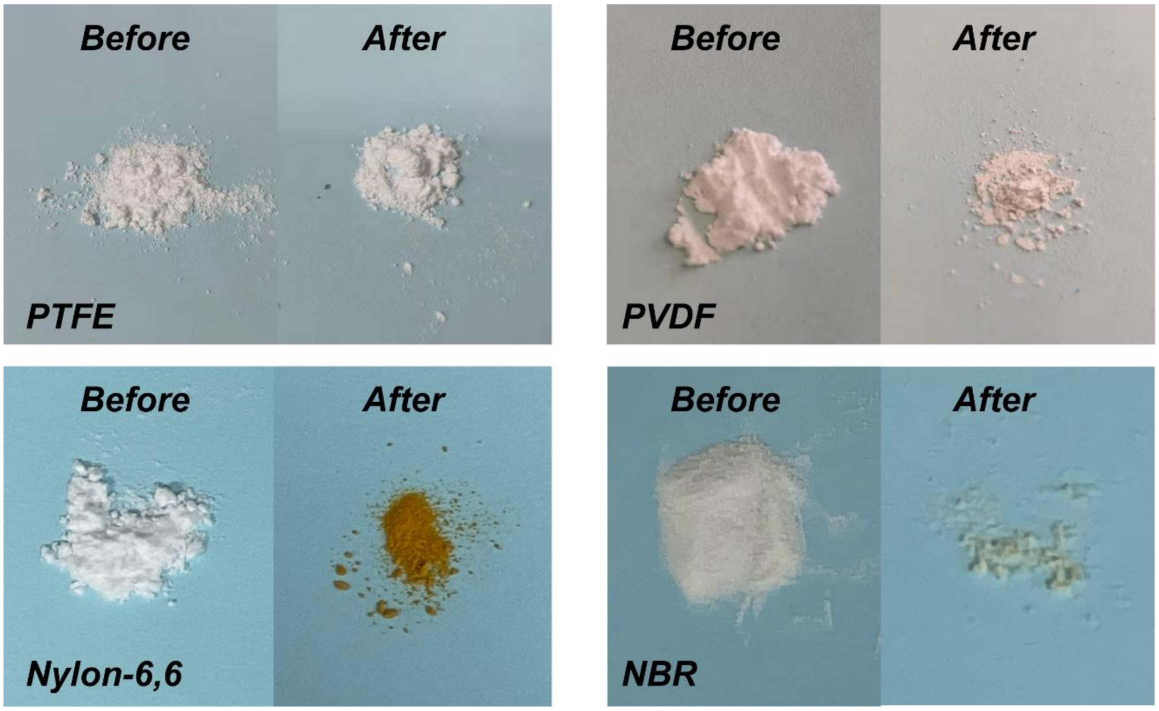

Supplementary Fig. 4 | Photographs of various dielectric powders before and after degradation of MO. (PTFE and PVDF are negatively charged powders, while Nylon-6,6 and NBR are positively)

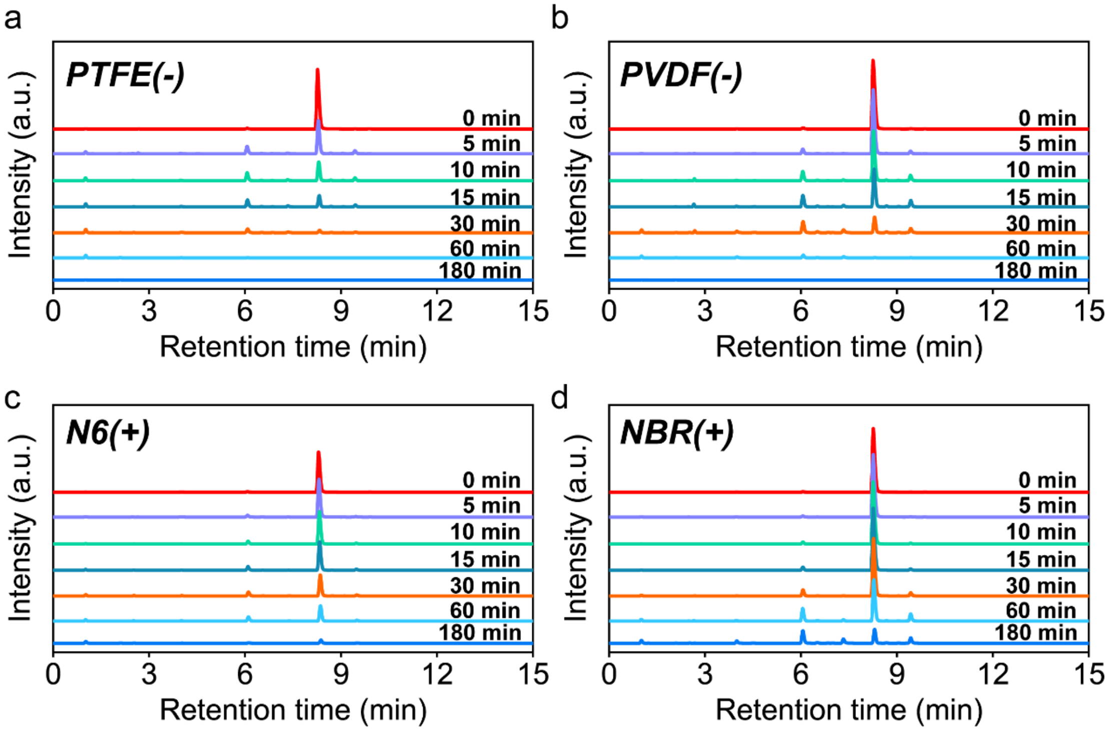

Supplementary Fig. 5 | Mass spectra of a 5-ppm MO solution during degradation by various powders. a, PTFE. **b,** PVDF. **c,** Nylon-6,6. **d,** NBR-Rubber**.**

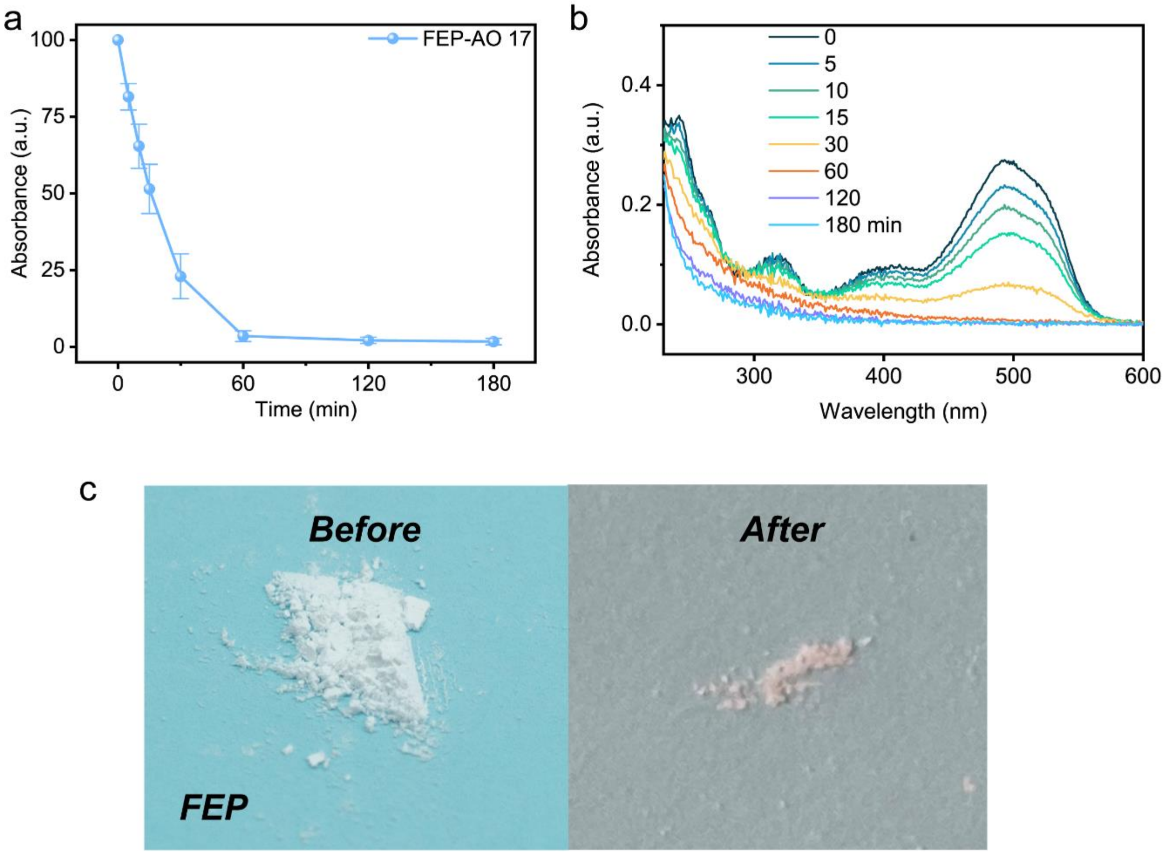

Supplementary Fig. 6 | Investigation on the discoloration of Acid Orange 17 (AO-17) by CEC using FEP powders. a, Evolution of the absorbance of a 5ppm AO-17 solution during ultrasonication. **b,** UV-Vis spectra of AO17 solution specific time intervals. **c,** Photographs of the FEP powder before and after the discoloration of an AO-17 solution. Error bars represent standard deviation based on three replicate data.

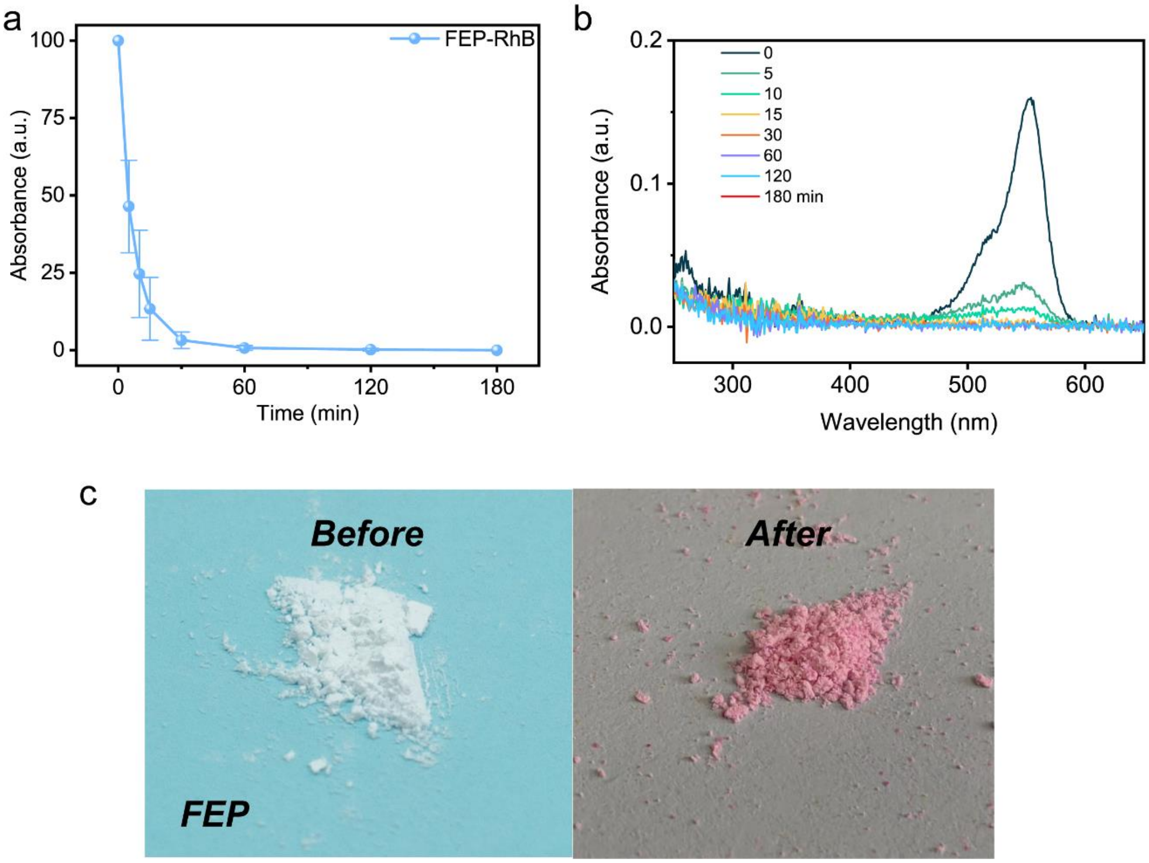

Supplementary Fig. 7 | Investigation on the discoloration of Rhodamine B (RhB) by CEC using FEP powders. a, Evolution of the absorbance of a 5ppm RhB solution during ultrasonication. **b,** UV-Vis spectra of RhB solution at specific time intervals. **c,** Photographs of the FEP powder before and after the discoloration of a RhB solution. Error bars represent standard deviation based on three replicate data.

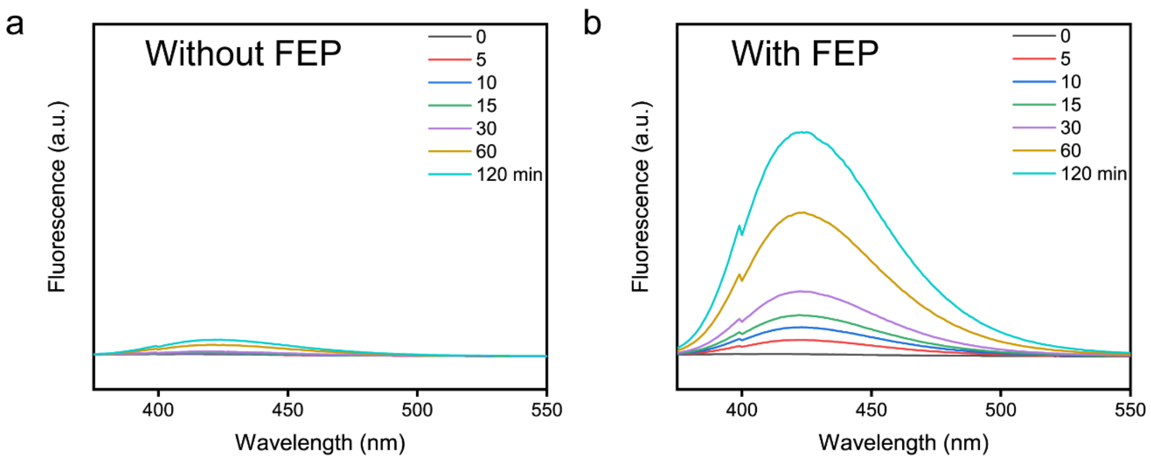

Supplementary Fig. 8 | Investigations on the generation of reactive hydroxyl radicals. Fluorescence spectra of THA-OH at different ultrasonication time in absence **(a)** and in presence **(b)** of FEP particles.

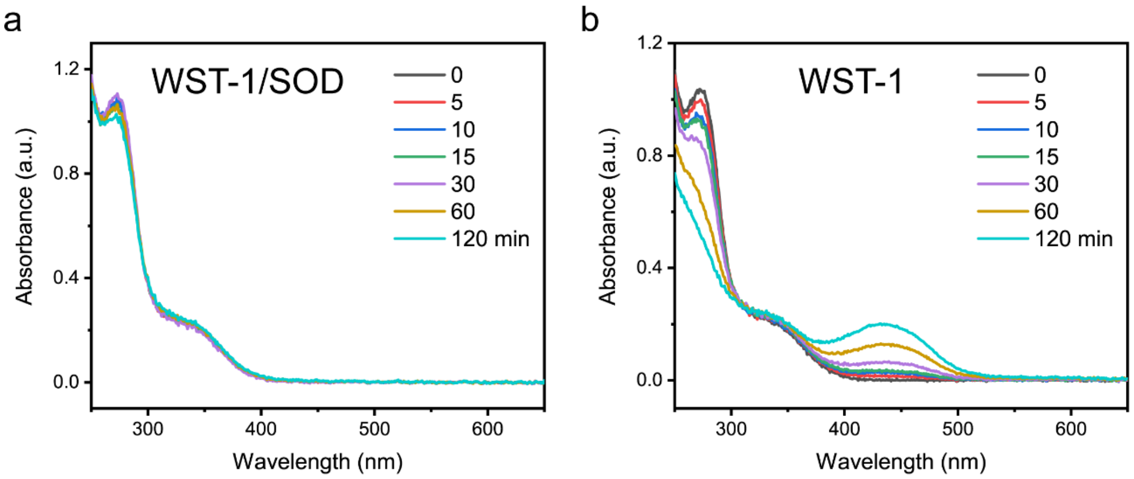

Supplementary Fig. 9 | Investigations on the generation of reactive superoxide radicals. UV-Vis spectra of WST-1 at different ultrasonication time in presence of FEP particles with (**a**) or without (**b**) addition of superoxide dismutase (SOD).

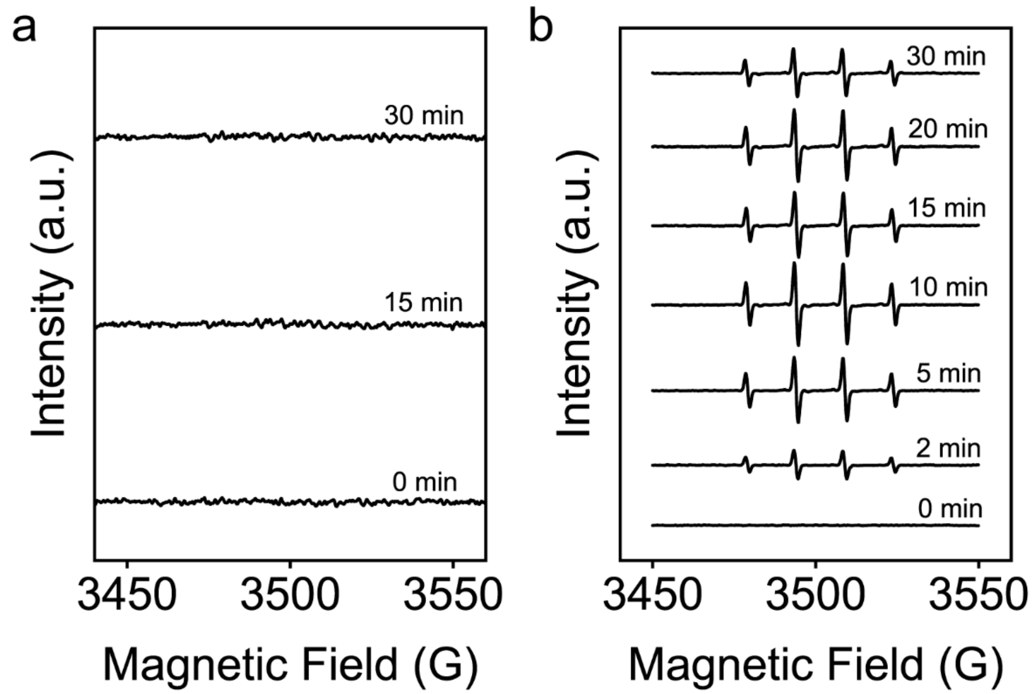

Supplementary Fig. 10 | Evolution of EPR diagrams in absence/ presence of FEP powders under ultrasonication. a, without FEP. **b,** with FEP.

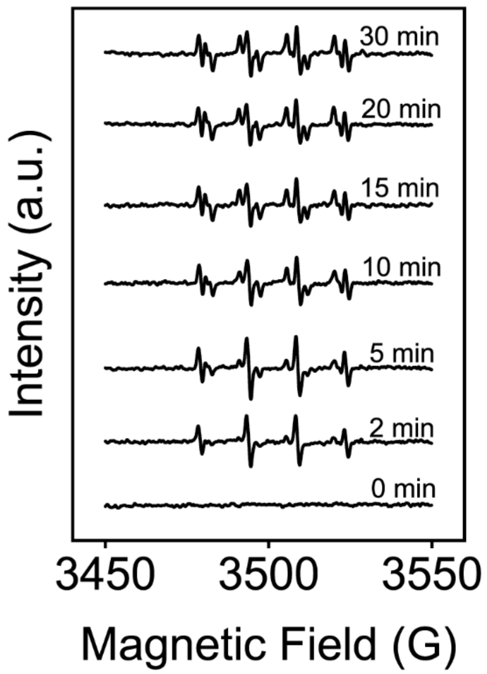

Supplementary Fig. 11 | Evolution of EPR diagrams after introducing 1 mM ter-butanol.

Supplementary Table 1. Comparisons between representative tribocatalysis literatures and this work

Added materials	Features	Condition	Principle	Ref.
$BiOIO_3$	Ferroelectric	Magnetic Stir	Tribocatalysis	9
Bi_2WO_6	Piezoelectric	Magnetic Stir	Tribocatalysis	10
ZnO	Piezoelectric	Magnetic Stir	Tribocatalysis	11
$Ba_{0.75}Sr_{0.25}TiO_3$	Piezoelectric	Magnetic Stir	Tribocatalysis	12
CdS NWs	Piezoelectric	Magnetic Stir	Tribocatalysis	13
$Ba_{2.5}Sr_{2.5}Nb_8Ta_2O_{30}$	Ferroelectric	Magnetic Stir	Tribocatalysis	14
BaTiO3@Au	Piezoelectric	Magnetic Stir	Piezocatalysis	15
PZT	Piezoelectric	Magnetic Stir	Piezocatalysis	16
H-ZnS@SNG	Piezoelectric	Magnetic Stir	Piezocatalysis	17
Pristine polymers	Intrinsic catalytic inertness	Ultrasonication	Contact-Electro-Catalysis	This work

Supplementary Table 2. Main features of conventional catalysts for degrading pollutants under ultrasonication and their comparisons with this work.

Catalysts	Features	Pollutants	Condition	Ref.
$BaTiO_3$	Piezoelectric effect	4-Chlorophenol	110 W/40 kHz	18
$ZnO@TiO_2$	Piezo-photocatalytic effect	Methyl Orange	100 W UV/ 40kHz US	19
CuS/ZnO	Piezo-photocatalytic effect	Methylene Blue	500 W UV / 200 W US	20
Bi_2WO_6	Piezoelectric effect	Methyl Orange	80 W/40 kHz	21
$ZnSnO_3$	Piezoelectric effect	Rhodamine B	120 W/33 kHz	22
$BaZr_{0.02}Ti_{0.98}O_3$	Piezo-photocatalytic effect	Methyl Orange	24 W UV/70 W 40kHz US	23
$rGO/BiVO_4$	Piezoelectric effect	Methylene Blue	70 W/40 kHz	24
$BaTiO_3/Ag_2O$	Piezophototronic effect	Rhodamine B	50 W/40 kHz	25
$Ag@LiNbO_3/PVDF$	Piezoelectric effect	Methyl Orange	70 W/40 kHz	26
Pristine polymers	Intrinsic catalytic inertness	Methyl Orange	120 W/40 kHz	This work

Supplementary References

1. Stoll, S. & Schweiger, A. EasySpin, a comprehensive software package for spectral simulation and analysis in EPR. *J. Magn. Reason.* **178**, 42-55 (2006).
2. Perdew, J. P., Burke, K. & Ernzerhof, M. Generalized gradient approximation made simple. *Phys. Rev. Lett.***77**, 3865-3868 (1996).
3. Caruso, F. et al. First-principles description of charge transfer in donor-acceptor compounds from self-consistent many-body perturbation theory. *Phys. Rev. B* **90,** 085141 (2014).
4. Delley, B. From molecules to solids with the DMol 3 approach. *J. Chem. Phys.* **113**, 7756-7764 (2000).
5. Delley, B. An all-electron numerical method for solving the local density functional for polyatomic molecules. *J. Chem. Phys.* **92**, 508-517 (1990).
6. Dolg, M., Wedig, U., Stoll, H. & Preuss, H. Energy-adjusted abinitio pseudopotentials for the first row transition elements. *J. Chem. Phys.* **86**, 866-872 (1987).
7. Bergner, A., Dolg, M., Küchle, W., Stoll, H. & Preuß, H. Ab initio energy-adjusted pseudopotentials for elements of groups 13–17. *Mol. Phys.* **80**, 1431-1441 (1993).
8. Wang, Y. et al. Ultrasonic activation of inert poly (tetrafluoroethylene) enables piezocatalytic generation of reactive oxygen species. *Nat. Commun.* **12**, 1-8 (2021).
9. Lei, H., et al. Tribo-catalytic degradation of organic pollutants through bismuth oxyiodate triboelectrically harvesting mechanical energy. *Nano Energy* **78**, 105290 (2020).
10. Wu, M., Lei, H., Chen, J. & Dong, X. Friction energy harvesting on bismuth tungstate catalyst for tribocatalytic degradation of organic pollutants. *J. Colloid Interface Sci.* **587**, 883-890 (2021).
11. Zhao, J., et al. Strong tribo-catalysis of zinc oxide nanorods via triboelectrically-harvesting friction energy. *Ceram. Int.* **46**, 25293-25298 (2020).
12. Li, P., et al. Strong tribocatalytic dye decomposition through utilizing triboelectric energy of barium strontium titanate nanoparticles. *Nano Energy* **63**, 103832 (2019).
13. Yang, B., et al. Enhanced tribocatalytic degradation using piezoelectric CdS nanowires for efficient water remediation. *J. Mater. Chem. C* **8**, 14845-14854 (2020).
14. Sun, C., et al. Tribocatalytic degradation of dyes by tungsten bronze ferroelectric Ba 2.5 Sr 2.5 Nb 8 Ta 2 O 30 submicron particles. *RSC Adv.* **11**, 13386-13395 (2021).
15. Liu, X., et al. Low frequency hydromechanics-driven generation of superoxide radicals via optimized piezotronic effect for water disinfection. *Nano Energy* **88**, 106290 (2021).
16. Feng, Y., et al. Engineering spherical lead zirconate titanate to explore the essence of piezo-catalysis. *Nano Energy* **40**, 481-486 (2017).
17. Pan, M., Liu, S., Pan, B. & Chew, J. W. Directionally tailoring the macroscopic polarization of piezocatalysis for hollow zinc sulfide on dual-doped graphene. *Nano Energy* **88**, 106312 (2021).
18. Lan, S., et al. Performance and mechanism of piezo-catalytic degradation of 4-chlorophenol: finding of effective piezo-dechlorination. *Environ. Sci. Technol.* **51**, 6560-6569 (2017).
19. You, H., et al. High-efficiency and mechano-/photo-bi-catalysis of piezoelectric-ZnO@ photoelectric-TiO2 core-shell nanofibers for dye decomposition. *Chemosphere* **183**, 528-535 (2017).
20. Hong, D., et al. High piezo-photocatalytic efficiency of CuS/ZnO nanowires using both solar and mechanical energy for degrading organic dye. *ACS Appl. Mater. Interfaces* **8**, 21302-21314 (2016).
21. Kang, Z., et al. Effect of Bi2WO6 nanosheets on the ultrasonic degradation of organic dyes: Roles of adsorption and piezocatalysis. *J. Cleaner Prod.* **261**, 121125 (2020).
22. Biswas, A., Saha, S. & Jana, N. R. ZnSnO3 nanoparticle-based piezocatalysts for ultrasound-assisted degradation of organic pollutants. *ACS Appl. Nano Mater.* **2**, 1120-1128 (2019).
23. Sharma, M., Singh, G. & Vaish, R. Dye degradation and bacterial disinfection using

multicatalytic BaZr0. 02Ti0. 98O3 ceramics. *J. Am. Ceram. Soc.* **103**, 4774-4784 (2020).
24. Kumar, M., Singh, G. & Vaish, R. A reduced graphene oxide/bismuth vanadate composite as an efficient piezocatalyst for degradation of organic dye. *Mater. Adv.*, (2021).
25. Li, H., et al. Enhanced Ferroelectric-Nanocrystal Based Hybrid Photocatalysis by Ultrasonic Wave Generated Piezo-phototronic Effect. *Nano Lett.* **15**, 2372-2379 (2015).
26. Singh, G., Sharma, M. & Vaish, R. Flexible Ag@ LiNbO3/PVDF Composite Film for Piezocatalytic Dye/Pharmaceutical Degradation and Bacterial Disinfection. *ACS Appl. Mater. Interfaces* **13**, 22914-22925 (2021).
