# Supplementary material for: Contact-electro-catalysis for the degradation of organic pollutants using pristine dielectric powders
Source: Nat Commun. 2022 Jan 10;13:130. doi: 10.1038/s41467-021-27789-1 (PMC8748705; doi:10.1038/s41467-021-27789-1)
Supplement: Supplementary file 2 — Description of Additional Supplementary Files [file 41467_2021_27789_MOESM2_ESM.pdf]

Description of Additional Supplementary Files

File Name: Supplementary Movie 1
Description: Video demonstration of degrading MO solution in presence of pristine FEP powder, and its comparison to without FEP
